# Heat Shock-Related Protein Responses and Inflammatory Protein Changes Are Associated with Mild Prolonged Hypoglycemia

**DOI:** 10.3390/cells10113109

**Published:** 2021-11-10

**Authors:** Abu Saleh Md Moin, Manjula Nandakumar, Hassan Kahal, Thozhukat Sathyapalan, Stephen L. Atkin, Alexandra E. Butler

**Affiliations:** 1 Diabetes Research Center (DRC), Qatar Biomedical Research Institute (QBRI), Hamad Bin Khalifa University (HBKU), Qatar Foundation (QF), P.O. Box 34110, Doha 5825, Qatar; amoin@hbku.edu.qa (A.S.M.M.); mnandakumar@hbku.edu.qa (M.N.); 2 Academic Endocrinology, Diabetes and Metabolism, Hull York Medical School, Hull YO10 5DD, UK; hassan.kahal@yahoo.co.uk (H.K.); Thozhukat.Sathyapalan@hyms.ac.uk (T.S.); 3 Royal College of Surgeons of Ireland, Busaiteen P.O. Box 15503, Bahrain; satkin@rcsi.com

**Keywords:** type 2 diabetes, hypoglycemia, heat shock proteins, oxidative stress, inflammatory proteins

## Abstract

Mild hypoglycemia is common in clinical practice. Severe hypoglycemia results in heat shock protein and associate co-chaperone changes. Whether mild prolonged hypoglycemia elicits a similar response with inflammatory and oxidative-stress responses compared with a severe hypoglycemic event is unclear; therefore, this pilot exploratory study was undertaken. We performed a case–control induced hypoglycemia clamp study, maintaining blood glucose at 2.8 mmol/L (50 mg/dL) for 1 h in 17 subjects (T2D (*n* = 10); controls (*n* = 7)). Blood sampling was performed at baseline, hypoglycemia, and 24 h; slow off-rate modified aptamer (SOMA)-scan plasma protein analysis of HSP-related proteins, inflammatory stress markers, and oxidative stress markers was performed. In total, 16 HSPs were analyzed. At baseline, TLR4:MD-2 complex was elevated (*p* = 0.01), whilst HSPA8 was lower (*p* < 0.05) in T2D. At hypoglycemia, UBE2N, STIP1, and UBE2L3 increased (all *p* < 0.05), whilst TLR4:MD-2 and HSPA8 decreased (*p* < 0.05) in T2D versus baseline. In follow-up after hypoglycemia, HSPs normalized to baseline by 24 h, except UBE2L3 (*p* < 0.05), which was decreased in controls versus baseline. Correlation of altered inflammatory markers with HSPs revealed the following: at baseline, TLR4:MD-2 correlated with CXCL10 (*p* < 0.01) and SIGLEC1 (*p* < 0.05) in controls; HSPA8 negatively correlated with IL5 (*p* < 0.05) in T2D. A negative correlation between urinary isoprostane 8-iso PGF2α, a marker of oxidative stress, and HSPA1A was seen at 24 h in T2D only (*p* < 0.05). In conclusion, the HSP changes seen for mild prolonged hypoglycemia were similar to those previously reported for a severe event. However, mild prolonged hypoglycemia appeared to elicit an increased inflammatory response that was associated with heat shock and related proteins.

## 1. Background

Optimal management of type 2 diabetes (T2D) with stricter glucose control increases the risk and frequency of hypoglycemic episodes [1], and hypoglycemia has been directly linked to diabetes-related complications such as cognitive dysfunction [2]. Diabetes progression and the development of diabetes-related complications have been associated with intracellular protein misfolding [3,4]. Heat shock proteins (HSPs) help to maintain cellular homeostasis and prevent the endoplasmic reticulum stress that results from protein misfolding through endogenous or exogenous inflammatory insults and oxidative stress generation [5]. HSPs are constitutively expressed and rapidly respond to cellular stress [5] and may be post-translationally modified including phosphorylation, acetylation, ubiquitination, AMPylation, and ADP-ribosylation that may modulate their function [6,7,8].

**Figure 1:**
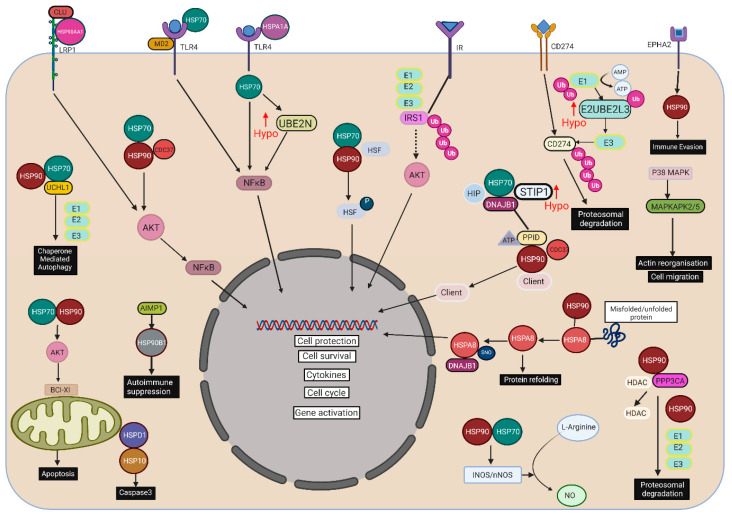
Schematic representing the recognized interactions between heat shock proteins (HSPs) and related proteins that, in response to severe acute hypoglycemia, are differentially expressed. Upward red arrows show proteins that, in T2D, were upregulated in response to hypoglycemia. HSP90 alpha (HSP90AA1, HSP90AB1, HSP90 beta, HSP90 dimer); HSPA1A, heat shock 70 kDa protein 1A; HSPA8, heat shock cognate 71 kDa protein; HSPD1, 60 kDa heat shock protein, mitochondrial; AIMP1, aminoacyl tRNA synthase complex-interacting multifunctional protein 1; CDC37, Hsp90 co-chaperone Cdc37; CLU, clusterin; DNAJB1, DnaJ homolog subfamily B member 1; MAPKAPK2, MAP kinase-activated protein kinase 2; MAPKAPK5, MAP kinase-activated protein kinase 5; PPID, peptidyl-prolyl cis-trans isomerase D; PPP3CA, serine/threonine-protein phosphatase 2B catalytic subunit alpha isoform; STIP1, stress-induced phosphoprotein 1; TLR4, toll-like receptor 4; TLR4:MD-2 complex, toll-like receptor 4 in complex with MD-2; CD274, programmed cell death 1 ligand 1; EPHA2, ephrin type-A receptor 2; E1, ubiquitin-activating enzyme; E2, ubiquitin-conjugating enzyme 2 (UBE2L3, ubiquitin-conjugating enzyme E2 L3; UBE2N, ubiquitin-conjugating enzyme E2 N); UCHL1, ubiquitin carboxyl-terminal hydrolase isozyme L1; E3, ubiquitin ligases; NFκB, nuclear factor kappa-light-chain-enhancer of activated B cells; AKT, protein kinase B, HSF, heat shock factors; SNO, S-nitrosylation; P38 MAPK, p38 mitogen-activated protein kinases; Bcl-xL, B-cell lymphoma-extra large; LRP1, low-density lipoprotein receptor-related protein 1; IR, insulin receptor; IRS1, insulin receptor substrate 1.

Proteins that are damaged or misfolded and that may result in cellular catastrophe are chaperoned by HSPs to the ubiquitin proteasome system (UPS) that functions as the main mechanism for proteolysis, effecting degradation of short-lived, damaged, or misfolded proteins [4]. The UPS is coordinated sequentially by three enzymes: ubiquitin-activating enzyme (E1), ubiquitin-conjugating enzyme (E2), and ubiquitin-protein ligase (E3); following ubiquitination, the 26S proteasome effects proteolysis [9] (Figure 1). HSPs are involved in binding and modulation of several critical enzymes involved in inflammation, apoptosis, metabolism, and cell signaling [10]. HSPs are implicated in β-cell dysfunction and insulin resistance [11], as well as playing a role in diabetes complications. Diabetic neuropathy and nephropathy are associated with elevated HSP27 [12], whilst HSP70 has been associated with diabetic retinopathy [13] and is inversely related to macrovascular complications [13].

Oxidative stress results in damage of proteins and DNA [14], has been associated with both microvascular and macrovascular diabetic complication development [15,16], and is integral to vascular inflammation resulting in vascular damage [17]. Oxidative stress results when free radical generation is excessive and/or the normal defense mechanisms are compromised [18]; the inflammation generated leads to further oxidative stress, thus perpetuating the negative effects [19,20]. We have shown previously that hypoglycemia results in platelet activation and inflammatory and oxidative stress responses [21,22] with dysregulated proteomic pathways in diabetes [23].

Glucose modulation is associated with changes in HSPs [24], and severe hypoglycemia has been associated with HSP changes at baseline that exhibit an exaggerated response to hypoglycemia associated with changes in inflammatory markers [25]. We hypothesized that mild, although prolonged, hypoglycemia would have less effect than severe transient hypoglycemia on the HSP and related protein responses and the associated inflammatory and oxidative changes; hence, this pilot exploratory study was performed.

## 2. Methods

This prospective case-control study enrolled adult T2D subjects (*n* = 10) and control subjects (*n* = 7); apart from one individual who was South Asian, all were Caucasian; all were in the age range 40–53 years. A hypoglycemic clamp, lowering blood glucose to 2.8 mmol/L (50 mg/dL for 1 h duration was performed. Blood sampling was undertaken at the following timepoints: baseline, hypoglycemia, 24 h post hypoglycemia [21].

The study was approved by the Yorkshire and the Humber Research Ethics Committee, registered at www.clinicaltrials.gov (accessed on 1 August 2021) (NCT02205996), and performed from November 2011 to May 2013 in the Diabetes Centre, Hull Royal Infirmary. Written informed consent was provided by all participants. To be included in the T2D cohort, a diabetes duration <10 years was required together with the subjects being on a stable medication regime and dose for the preceding 3 months (statin, metformin, and/or angiotensin-converting enzyme inhibitor/angiotensin receptor blocker were allowed; no glucose lowering medications besides metformin were allowed). Further inclusion criteria included levels of HbA1c < 10% (86 mmol/mol), no past medical history of hypoglycemia or hypoglycemic unawareness in the preceding 3-months. For control participants, an oral glucose tolerance test was used to exclude diabetes. Body mass index (BMI) for all participants was in the range of 18 to 49 kg/m^2^; other inclusion criteria were normal renal and liver biochemistry; no past medical history of cancer; no contraindication to insulin infusion to reach hypoglycemia such as ischemic heart disease, epilepsy, seizure history, adrenal insufficiency, drop attacks or treated hypothyroidism). Prior to enrolment, participants underwent a medical history, physical examination, an electrocardiogram and routine blood tests.

### 2.1. Biochemical Markers

Centrifugation (2000× *g*, 15 min, 4 °C) of blood samples was undertaken; storage of aliquots at −80 °C was done within 30 min of collection, awaiting batch analysis. Triglycerides, total cholesterol and fasting plasma glucose (FPG), and were measured using a Beckman AU 5800 analyzer (Beckman-Coulter, High Wycombe, United Kingdom) [21,26]. Urinary isoprostane 8-iso PGF2α was measured as a marker of oxidative stress; this was accomplished using a urinary isoprostane EIA kit (Oxford Biomedical Research, Oxford, United Kingdom) following the manufacturer’s instructions [22].

### 2.2. SOMA-Scan Assay and Statistical Analysis

Plasma proteins were quantified using the SOMAscan assay that utilized a Tecan Freedom EVO liquid handling system (Tecan Group, Maennedorf, Switzerland) and assays were performed following the manufacturer’s instructions [27,28].

### 2.3. Data Processing and Analysis

Initial relative fluorescent units (RFUs) were aquired utilizing the using Agilent Feature Extraction Software V12.0 (Agilent, Santa Clara, CA, USA) with their normalisation and calibration performed using the SomaLogic software.

No studies are available detailing the changes in HSP response to hypoglycemia on which a power calculation could be based. log_2_ RFU values were derived using R version 3.5.2 (R Foundation for Statistical Computing, Vienna, Austria), as documented previously [29]. Protein changes were determined using limma models that contained contrasts between timepoints, as well as contrasts between groups at single timepoints. The Benjamini–Hochberg method was used to correct limma-obtained *p*-values [30]. A *p*-value of <0.05 was considered to be significant. A visual evaluation of data trends was undertaken. The Kolmogorov–Smirnov test was used to identify non-normal data and non-parametric tests were then applied. Student’s *t*-test was used to compare data at each timepoint between groups and between timepoints within groups. GraphPad Prism (San Diego, CA, USA) was used for statistical analysis [27,28].

### 2.4. Protein–Protein Interaction Tools

STRING 11.0 (Search Tool for the Retrieval of Interacting Genes; The Swiss Institute of Bioinformatics, Lausanne, Switzerland) was used to visualize the defined as well as the predicted protein–protein interactions for key HSP and inflammatory proteins (https://string-db.org/ (accessed on 1 September 2021)).

Ingenuity Pathway Analysis (IPA) software version 8.7 (Qiagen, Germantown, MD, USA) was used to illustrate the pathways related to key HSP and inflammatory proteins presented in this study.

## 3. Results

### 3.1. Study Participants

Demographic and biochemical data for the 17 control (*n* = 7) and T2D (*n* = 10) subjects included in the study are shown in Table 1.

Levels of urinary isoprostane 8-iso PGF2α, a marker of oxidative stress, have been reported previously in these subjects, where an increase was seen from baseline to 24 h in both controls and T2D; however, this only reached significance in the T2D group (*p* = 0.02) (Table 1) [22].

**Figure 2:**
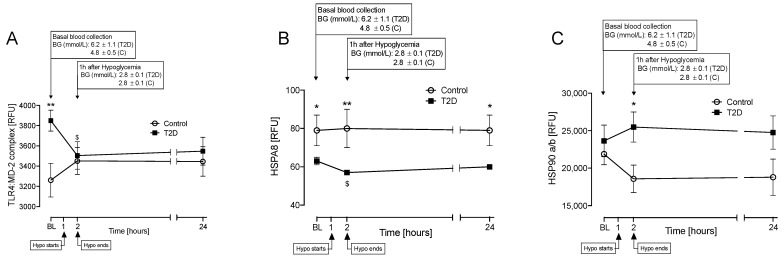
Comparison of plasma levels of the following heat shock proteins: toll-like receptor 4:myeloid differentiation factor-2 (TLR4:MD-2), heat shock protein family A (HSP70) member 8 (HSPA8), and heat shock protein 90 dimer (HSP90a/b) at baseline (BL), at hypoglycemia and 24 h post-hypoglycemia in control and T2D cohorts. Controls (white circles), T2D (black squares). TLR4:MD-2 (**A**), HSPA8 (**B**), and HSP90a/b (**C**). Panels (**A**,**B**) show proteins for which levels differed at BL between T2D and control cohorts. Statistics: *, *p* < 0.05; **, *p* < 0.01, control vs. T2D; ^$^, *p* < 0.05, T2D BL vs. Hypo. RFU, relative fluorescent units; Hypo, hypoglycemia.

**Figure 3:**
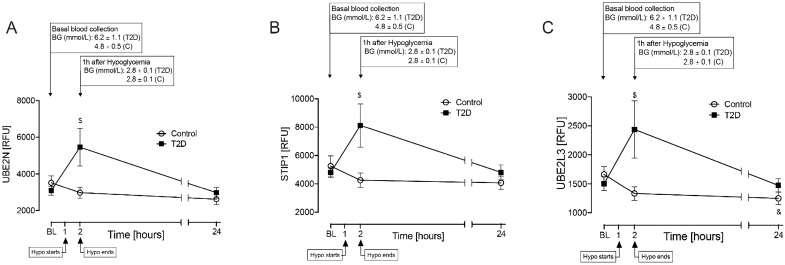
Comparison of plasma levels of the following heat shock proteins: ubiquitin-conjugating enzyme E2N (UBE2N), stress-induced phosphoprotein 1 (STIP1), and ubiquitin-conjugating enzyme E2L3 (UBE2L3) at baseline (BL), at hypoglycemia, and 24 h post hypoglycemia in control and T2D cohorts. Controls (white circles), T2D (black squares). UBE2N (**A**), STIP1 (**B**), and UBE2L3 (**C**), proteins for which levels differed from baseline to hypoglycemia in T2D and control cohorts. Statistics: ^$^, *p* < 0.05, T2D BL vs. Hypo; ^&^, *p* < 0.05, control BL vs. 24 h. RFU, relative fluorescent units; Hypo, hypoglycemia.

A total of 16 HSP-related proteins were identified, of which there were changes in 6 (Figure 2 and Figure 3) and no changes seen for 10 (Supplementary Figure S1). The proteins analyzed were toll-like receptor (TLR) 4 and myeloid differentiation factor 2 (TLR4:MD-2) complex; heat shock cognate 71 kDa protein (HSPA8); heat shock protein 90 alpha/beta dimer (HSP90a/b); ubiquitin-conjugating enzyme E2 N (UBE2N); stress-induced phosphoprotein 1 (STIP1); ubiquitin-conjugating enzyme E2L 3 (UBE2L3); ubiquitin carboxyl-terminal hydrolase isozyme L1 (UCHL1); calcineurin; MAP kinase-activated protein kinase 5 (MAPKAPK5); MAP kinase-activated protein kinase 2 (MAPKAPK2); heat shock protein family D (Hsp60) member 1 (HSPD1); heat shock 70 kDa protein 1A (HSPA1A); DnaJ homolog subfamily B member 1 (DNAJB1); clusterin (CLU); Hsp90 co-chaperone Cdc37 (CDC37); and programmed cell death 1 ligand 1 (CD274).

### 3.2. Proteins That Differed between T2D and Control at Baseline and at Hypoglycemia

At baseline, TLR4:MD-2 complex was higher (*p* = 0.01), whilst HSPA8 was lower (*p* < 0.05) in T2D (Figure 2A,B).

At hypoglycemia, TLR4:MD-2 complex and HSPA8 both decreased in T2D versus baseline (*p* < 0.05) (Figure 2A,B), with HSPA8 remaining significantly lower at hypoglycemia in T2D versus controls (*p* < 0.01). HSP90a/b increased in T2D at hypoglycemia compared to control (*p* < 0.05; Figure 2C). At hypoglycemia, UBE2N, STIP1, and UBE2L3 increased in T2D compared to baseline (*p* < 0.05, *p* < 0.05, and *p* < 0.05, respectively), a change not seen in controls (Figure 3A–C).

In the follow-up period after hypoglycemia, HSPA8 remained lower in T2D (*p* < 0.05; Figure 2B), UBE2L3 (*p* < 0.05) was decreased in controls compared to baseline (Figure 3C), and the other HSPs normalized to baseline by 24 h.

Ten HSPs, namely, UCHL1, calcineurin, MAPKAPK5, MAPKAPK2, HSPD1, HSP1A, DNAJB1, clusterin, CDC37, and CD274, did not differ at any time point (Supplementary Figure S1).

Correlations were made between the HSPs and the 15 inflammation and 1 oxidative stress markers that, as previously reported [21], differed with hypoglycemia. These are shown in Supplementary Table S1: C-X-C motif chemokine 10 (CXCL10), interleukin-5 (IL5), azurocidin (AZU1), C-type lectin domain family 7 member A (CLEC7A), serine/threonine-protein kinase (TBK1), protein kinase C zeta type (PRKCZ), ribosomal protein S6 kinase alpha-5 (RPS6KA5), CD40 ligand (CD40LG), interleukin-34 (IL34), high mobility group protein B1 (HMGB1), protein S100-A9 (S100A9), interleukin-1 beta (IL1B), C-C motif chemokine 19 (CCL19), sialoadhesin (SIGLEC1), and interleukin 10 receptor beta subunit (IL10RB) [21].

### 3.3. Correlations between Inflammatory and Oxidative Stress Markers for HSPs That Differed Significantly between T2D and Control at Baseline (TL4:MD-2 Complex and HSPA8)

Correlations at baseline of the 15 inflammation and one oxidative stress markers with the HSPs that have been shown to differ at baseline between T2D and controls [21].

**Figure 4:**
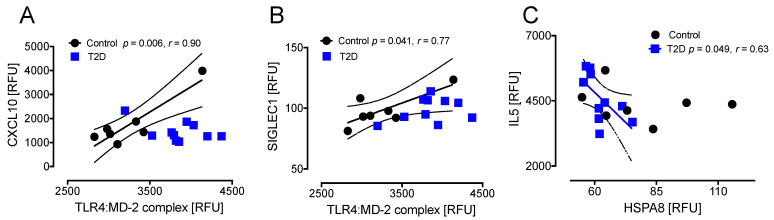
Correlations of HSPs that differed at baseline between T2D and control subjects with inflammatory proteins. At baseline, TLR4:MD-2 complex showed a positive correlation with CXCL10 (**A**) and SIGLEC1 (**B**) in control subjects. HSPA8 showed a negative correlation with IL5 (**C**) in T2D subjects. TLR4:MD-2 complex, toll-like receptor 4 in complex with MD-2; HSPA8, heat shock cognate 71 kDa protein; IL5, interleukin 5; RFU, relative fluorescent units.

TLR4:MD-2 correlated positively with CXCL10 (r = 0.9, *p* < 0.006) and SIGLEC1 in controls (r = 0.77, *p* < 0.04) (Figure 4A,B). HSPA8 negatively correlated with IL5 in T2D (r = 0.63, *p* < 0.05) (Figure 4C).

### 3.4. Correlations between Inflammatory and Oxidative Stress Markers for HSPs (UBE2N and STIP1) and UBE2L3 That Differed Significantly between T2D and Control at Hypoglycemia

**Figure 5:**
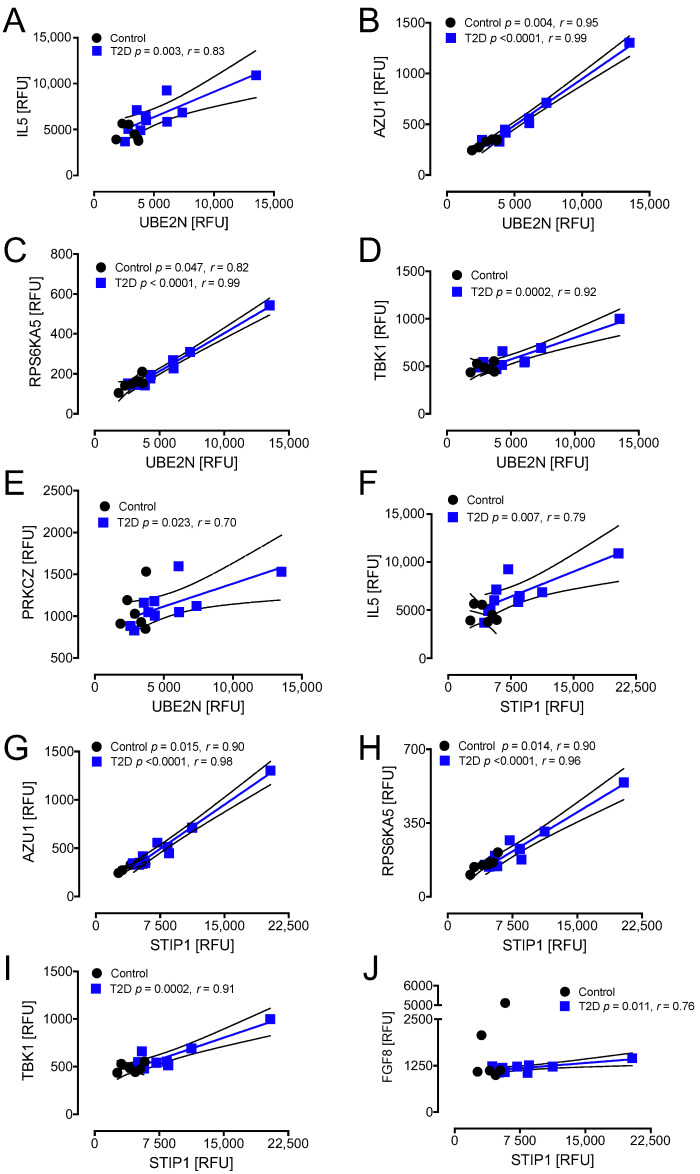
Correlations of HSPs that changed significantly from baseline to hypoglycemia in T2D with inflammatory markers at hypoglycemia. At hypoglycemia, UBE2N showed a positive correlation with IL5 (**A**), AZU1 (**B**), RPS6KA5 (**C**), TBK1 (**D**), and PRKCZ (**E**) in T2D subjects; UBE2N also had a positive correlation with RPS6KA5 (**C**) in control subjects. STIP 1 showed a positive correlation with IL5 (**F**), TBK1 (**I**), and FGF8 (**J**) in T2D subjects alone and with AZU1 (**G**) and RPS6KA5 (**H**) in both T2D and control subjects. UBE2N, ubiquitin-conjugating enzyme E2 N; STIP1, stress-induced-phosphoprotein 1; AZU1, azurocidin 1; RPS6KA5, ribosomal protein S6 kinase 5; TBK1, TANK-binding kinase 1; PRKCZ, protein kinase C zeta type; IL5, interleukin 5; FGF8, fibroblast growth factor 8; RFU, relative fluorescent units.

Correlations at hypoglycemia of the 15 inflammation and 1 oxidative stress markers with the HSPs that differed between T2D and control at hypoglycemia:

T2D: UBE2N correlated positively with IL5 (r = 0.83, *p* < 0.003), AZU1 (r = 0.99, *p* < 0.0001), RPS6KA5 (r = 0.99, *p* < 0.0001), TBK1 (r = 0.92, *p* < 0.001), and PRCKZ (r = 0.70, *p* < 0.05) in T2D. Controls: UBE2N correlated positively with AZU1 (r = 0.95, *p* < 0.01) and RPS6KA5 (r = 0.82, *p* < 0.05) in controls (Figure 5A–E).

T2D: STIP1 correlated positively with IL5 (r = 0.79, *p* < 0.01), AZU1 (r = 0.98, *p* < 0.0001), RPS6KA5 (r = 0.96, *p* < 0.0001), TBK1 (r = 0.91, *p* < 0.001), and FGF8 (r = 0.76, *p* < 0.05) in T2D. Controls: STIP1 correlated positively with AZU1 (r = 0.90, *p* < 0.05) and RPS6KA5 (r = 0.90, *p* < 0.05) in controls (Figure 5F–J).

**Figure 6:**
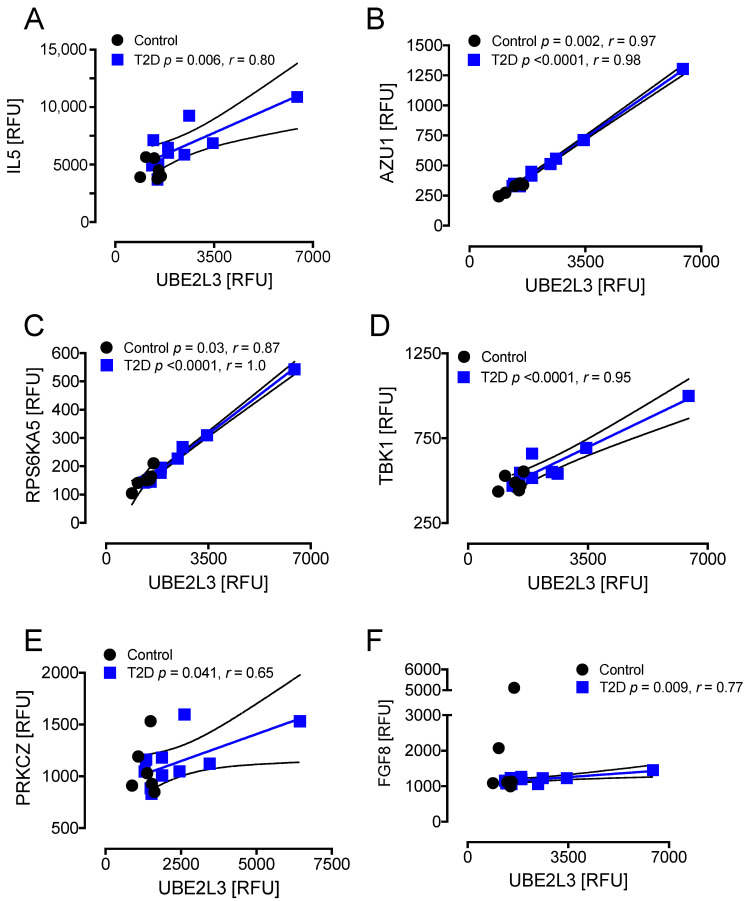
Correlations of HSPs that changed significantly from baseline to hypoglycemia in T2D with inflammatory markers at hypoglycemia. At hypoglycemia, UBE2L3 showed a positive correlation with IL5 (**A**), TBK1 (**D**), PRKCZ (**E**), and FGF8 (**F**) in T2D subjects only and with AZU1 (**B**) and RPS6KA5 (**C**) in both T2D and control subjects. UBE2L3, ubiquitin-conjugating enzyme E2L 3; IL5, interleukin 5; TBK1, TANK-binding kinase 1; PRKCZ, protein kinase C zeta type; FGF8, fibroblast growth factor 8; AZU1, azurocidin 1; RPS6KA5, ribosomal protein S6 kinase 5; RFU, relative fluorescent units.

T2D: UBE2L3 correlated positively with IL5 (r = 0.80, *p* < 0.01), AZU1 (r = 0.98, *p* < 0.0001), RPS6KA5 (r = 1.0, *p* < 0.0001), TBK1 (r = 0.95, *p* < 0.0001), PRCKZ (r = 0.65, *p* < 0.041), and FGF8 (r = 0.77, *p* < 0.01) in T2D (Figure 6A–F). Controls: UBE2L3 correlated positively with AZU1 (r = 0.97, *p* < 0.01) and RPS6KA5 (r = 0.87, *p* < 0.05) in controls (Figure 6B,C).

### 3.5. Correlations between Inflammatory and Oxidative Stress Markers for the HSP UBE2L3 That Differed Significantly between T2D and Controls at Baseline and at 24 h after Hypoglycemia

**Figure 7:**
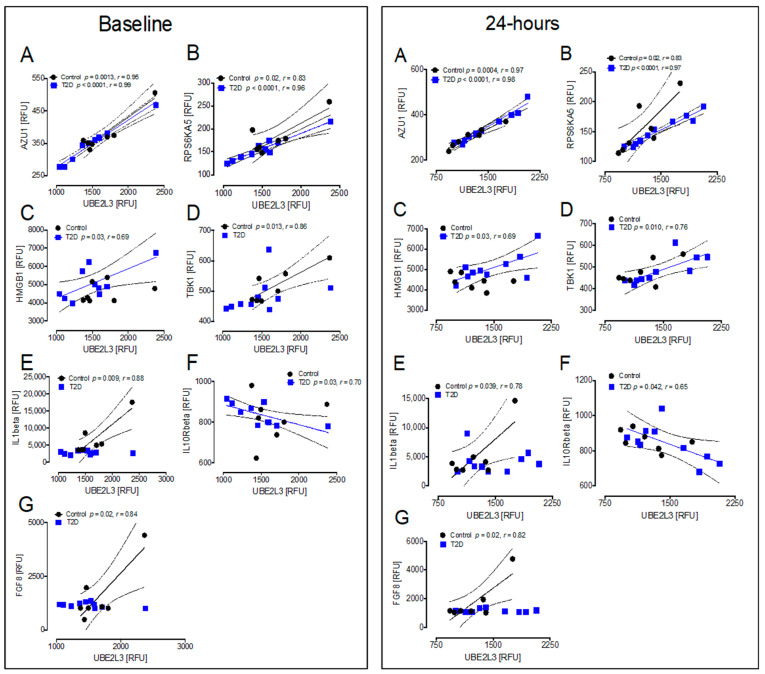
Comparison of the correlation of UBE2L3 with inflammatory markers at baseline and at 24 h. In T2D at 24 h, UBE2L3 correlated positively with AZU1 (**A**), RPS6KA5 (**B**), HMGB1 (**C**), and TBK1 (**D**), and negatively with IL10Rbeta (**F**). In controls at 24 h, UBE2L3 correlated positively with AZU1 (**A**), RPS6KA5 (**B**), IL1beta (**E**), and FGF8 (**G**). In comparison with the correlation of those markers at baseline, the same correlations were seen for both T2D and controls for AZU1 (**A**), RPS6KA5 (**B**), HMGB1 (**C**), IL1beta (**E**), IL10Rbeta (**F**), and FGF8 (**G**), and only differed for TBK1, which correlated in T2D at 24 h (**D**), but not at baseline, and did not correlate with controls at 24 h (**D**), despite correlating at baseline. UBE2L3, ubiquitin-conjugating enzyme E2L 3; AZU1, azurocidin 1; RPS6KA5, ribosomal protein S6 kinase 5; IL1beta, interleukin 1 beta: FGF8, fibroblast growth factor 8; HMGB1, high mobility group box 1; IL10Rbeta, interleukin 10 receptor beta; TBK1, TANK-binding kinase 1; RFU, relative fluorescent units.

Correlations at baseline and at 24 h for the 15 inflammation and 1 oxidative stress markers with UBE2L3: in T2D at 24 h, UBE2L3 correlated positively with AZU1 (r = 0.98, *p* < 0.0001), RPS6KA5 (r = 0.97, *p* < 0.0001), HMGB1 (r = 0.69, *p* < 0.03), and TBK1 (r = 0.76, *p* < 0.01), and negatively with IL10Rbeta (r = −0.65, *p* < 0.05) (Figure 7A–D,F). In controls at 24 h, UBE2L3 correlated positively with AZU1 (r = 0.97, *p* < 0.001), RPS6KA5 (r = 0.83, *p* < 0.05), IL1beta (r = 0.78, *p* < 0.05), and FGF8 (r = 0.82, *p* < 0.05) (Figure 7A,B,E,G). When comparing the correlations of those markers at 24 h with the correlations at baseline, the same correlations were seen for both T2D and controls for AZU1, RPS6KA5, HMGB1, IL1beta, IL10Rbeta, and FGF8 (Figure 7A–C,E–G), and only differed for TBK1, which correlated in T2D at 24 h but not at baseline, and did not correlate with controls at 24 h, despite correlating at baseline (Figure 7D).

### 3.6. Correlations between Urinary Isoprostane 8-Iso PGF2α and HSPs

Correlation of urinary isoprostanes with all of the HSPs included in this study was undertaken for both control and T2D subjects. At 24 h, HSPA1A correlated negatively with urinary isoprostane 8-iso PGF2α only in T2D subjects (r = 0.64, *p* = 0.046) (Supplementary Figure S2), but not with any of the other HSPs.

### 3.7. Interaction of HSPs by STRING Analysis

**Figure 8:**
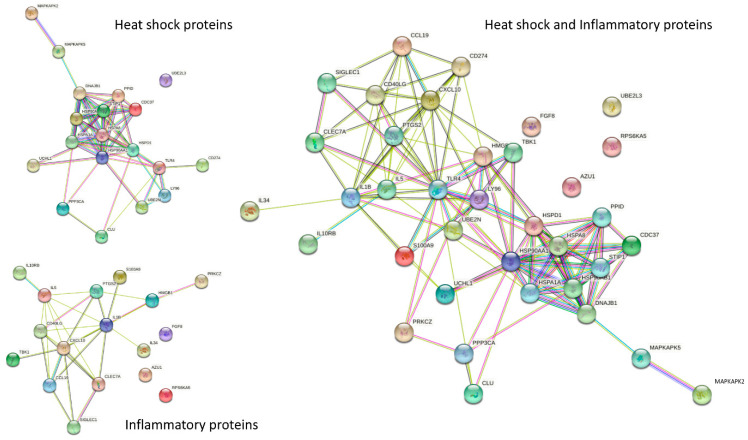
STRING interaction network showing the interactions of heat shock-related proteins. STRING 11.0 (Search Tool for the Retrieval of Interacting Genes) was used to visualize the recognized and predicted protein–protein interactions for the heat shock and inflammatory proteins reported here (https://string-db.org/ (accessed on 1 September 2021)). Network nodes represent proteins, and the lines reflect physical and/or functional interactions of proteins. Empty nodes represent the proteins of unknown three-dimensional structure, and filled nodes represent the proteins with some three-dimensional structure, either known or predicted. Different colored lines between the proteins represent the various types of interaction evidence in STRING (databases, experiments, neighborhood, gene fusion, co-occurrence, text mining, co-expression, and homology): here, known interactions are shown in light blue (from curated databases) and pink (experimentally determined); predicted interactions are shown in dark blue (gene co-occurrence); relationships gleaned from text mining (lime green), co-expression (black), and protein homology (mauve) are also shown. The heat shock proteins and the inflammatory proteins are shown separately (**left**) and combined (**right**).

The STRING protein interaction network demonstrates the close interactions of the heat shock-related proteins and between the inflammatory proteins (Figure 8). When the heat shock-related proteins and the inflammatory proteins were combined in the string analysis, it can be seen that there were interactions between them (Figure 8).

## 4. Discussion

These data show that baseline HSPs differed between T2D and control subjects, with further differences following hypoglycemia that correlated with inflammatory protein changes, suggesting that the inflammatory response may be driving the HSP changes. We have previously reported that inflammatory regulators are increased at the time of hypoglycemia, are exaggerated in T2D, and that all apart from C-reactive protein return to normal at 24 h [21]. These data are in accordance with the HSP response to transient severe hypoglycemia reported previously, although here there was a greater association between the HSP and the inflammatory responses [25].

The findings for HSPA8 and HSP90 were similar between severe [25] and mild hypoglycemia. The level of HSPA8 was lower in T2D at baseline, in accordance with those reporting lower HSP70 levels in diabetes [31], and decreased following hypoglycemia compared with controls. HSPA8 is part of the HSP70 family and acts as a molecular chaperone; its actions are facilitated by the HSP40 family and, in particular, the DNAJB proteins that, as shown here, were unaffected by hypoglycemia [32].

HSP90 did not differ at baseline but increased at hypoglycemia in T2D, suggesting that the hypoglycemic insult may have been of greater impact in T2D. HSP90 expression levels have been linked with T2D [33] and appeared not to be influenced by glucose levels during a euglycemic clamp [34].

Details of the response of the TLR4:MD-2 complex have not been reported before in hypoglycemia because, whilst the proteomic technology was the same [25], the proteomic panels differed slightly. TLR4:MD-2 complex was elevated in T2D and fell at hypoglycemia, although it did not return to baseline levels by 24-h. TLR4, a member of the TLR family, has an important role in modulating innate immunity [35]; activation of TLRs, by either exogenous or endogenous molecules, leads to a signaling cascade, resulting in cytokine production and induction of an adaptive immune response [36]. Myeloid differentiation protein-2 (MD2), a co-receptor of TLR4, is required for LPS recognition and activation of TLR4 [37]. Circulating levels of LPS, the classical TLR4 ligand, are higher in obese and T2D subjects and in rodent obesity/diabetes models, a scenario termed “metabolic endotoxemia” [38]. Consistent with our findings of elevated TLR4:MD-2 complex at baseline in T2D subjects, elevated expression of TLR4 has been associated with T2D [39], and elevated glucose has been reported to induce TLR2 and TLR4 expression in monocytes [40].

The UPS affects proteolysis, leading to degradation of short-lived, damaged, or misfolded proteins [4]. The UPS is coordinated sequentially by three enzymes: ubiquitin-activating enzyme (E1), ubiquitin-conjugating enzyme (E2), and ubiquitin-protein ligase (E3). In accord with the findings in severe hypoglycemia, there were significant increases in UBE2N, STIP1, and UBE2L3 seen in hypoglycemia only in the T2D cohort that returned to baseline by 24 h. UBE2L3 showed differences between controls and T2D at 24 h that reflected those seen in severe hypoglycemia [25]. UBE2N, a ubiquitin-conjugating enzyme E2; STIP1, an E3 ubiquitin ligase; and UBE2L3, also a ubiquitin ligase, are all key players in the ubiquitin pathway and critical for misfolded protein degradation [41]; our results suggest that, in T2D, hypoglycemia may be predisposed to increased protein misfolding, therefore activating this pathway. STIP1 has been shown to be upregulated and associated with attenuation of cell senescence by promoting ubiquitination [42], emphasizing its protective effect.

HSPs are implicated in both pro-inflammatory and anti-inflammatory responses [43], and it is not clear whether the resultant changes in HSPs seen here were a result of changes in the inflammatory and oxidative stress parameters, and that were correlated here, or vice versa. The inflammatory response in severe hypoglycemia was restricted to HSPA1A (HSP70), which correlated with IL-6 in the control subjects only [25]. Here, TLR4:MD-2 complex correlated positively with CXCL10 and SIGLEC1 in controls, whilst HSPA8 correlated negatively with IL5 in T2D. CXCL10 binds to its receptor, CXCR3, regulating immune responses through recruitment of leukocytes, such as T cells and monocytes/macrophages [44], and CXCL10 has been shown to bind with and activate toll-like receptor 4 (TLR4) [45]; this is in keeping with the positive correlation between TLR4:MD-2 complex and CXCL10 seen here at baseline in the control group, a relationship not found in the T2D group, likely because the levels of TLR4:MD-2 complex were already significantly elevated at baseline in T2D. SIGLEC1 has been reported to negatively regulate the TLR-4-mediated inflammatory response [46], in keeping with the positive correlation between SIGLEC1 and TLR4:MD-2 complex in the control group; again, this relationship was not seen in the T2D group. The role of IL5 with HSPA8 requires clarification.

An increase in urinary isoprostane in T2D at 24 h was seen, but it should be noted that the spot urine collection at 24 h would only reflect overnight urine production; however, the result is in accordance with the observations that the induced oxidative and inflammatory stress continued for a period after the event [25]. The correlation of urinary isoprostane with HSPA1A may be indicative of the relationship of oxidative stress measures with HSPs, but no other correlation with the HSPs was seen, perhaps due to a loss of any association between the blood and urinary measurement, the latter representing a global oxidative response.

The members of the UPS, UBE2N, STIP1, and UBE2L3, correlated with multiple inflammatory markers. IL5 stimulates protein ubiquitination [47]; ubiquitin-mediated autophagy receptors are phosphorylated by TBK1 (TANK-binding kinase 1) and ubiquitin ligase is an activator of TBK1 [48]. PRKCZ may associate with other proteins to form complexes that mediate ubiquitin-dependent degradation [49], whilst FGF8 function has been related to ubiquitin ligase action [50].

It should be noted that the correlation between the inflammatory proteins with UBE2L3, both prior to and at 24 h after the hypoglycemic insult, did not differ, indicating that the hypoglycemic insult had not disrupted these correlations. UBE2L3 ubiquitin conjugase is an indirect target of caspase-1 that proteolytically converts newly induced pro-interleukin 1 beta (IL-1β) into its mature form [51], thus explaining the changes in IL-1β seen in controls that appear to be lost in T2D. Overall, these multiple associations with mild though prolonged hypoglycemia were not seen in severe transient hypoglycemia [25], suggesting that the duration of the hypoglycemic insult was responsible for the changes in the inflammatory markers. Given that the changes in the HSPs were similar between severe transient and mild prolonged hypoglycemia, it is likely that the HSP changes are initiated and thus primed for any subsequent resulting inflammatory changes. The close association between the heat shock-related proteins was shown in the STRING analysis and, independently, also showed the close relationship between the inflammatory markers. When the two protein groups were combined in STRING, it was evident that close associations exist between the HSP and the inflammatory markers that reflect the associations reported here.

A strength of this study was the inclusion of T2D subjects of short diabetes duration with an age-matched healthy control group, and comparison with the severe hypoglycemic study was possible as the same proteomic platform was used [25]. The main study limitation was the small study numbers, given the invasive nature of the study, and a larger population may have shown a greater and even more integrated inflammatory protein and HSP response to hypoglycemia. T2D subjects were more obese than the controls, and it cannot be excluded that obesity may have contributed to the inflammatory response and HSP differences. The patients with T2D that were studied here were those on diet alone and on a stable dose of metformin, and it is unknown if a different therapeutic regimen would have affected their inflammatory and HSP responses. In addition, the measurement of HSP proteins may not reflect tissue level expression. As this was largely a Caucasian patient population, our findings may not be generalizable to other ethnic groups.

In conclusion, the HSP changes seen for mild prolonged hypoglycemia mirrored those previously reported for a severe event; however, mild prolonged hypoglycemia appeared to elicit an increased inflammatory response that was closely associated with heat shock-related proteins. Future studies are needed to validate and extend the findings presented here.

## Figures and Tables

**Table 1:** Demographic and biochemical parameters of control and type 2 diabetic (T2D) subjects included in the study [21]. Data are presented as mean ± 1 SD.

	**Controls (*n* = 7)**	**Type 2 Diabetes (*n* = 10)**
Age (years)	47 ± 6	46 ± 6
Sex (M/F)	4M/3F	7M/3F
BMI (kg/m^2^)	29 ± 4	36 ± 7
Systolic BP (mmHg)	126 ± 15	127 ± 20
Diastolic BP (mmHg)	75 ± 13	75 ± 11
Duration of diabetes (years)	N/A	3.3 ± 2.3
HbA1c (mmol/mol)	33.6 ± 2.9	49 ± 12
HbA1c (%)	5.2 ± 0.3	6.6 ± 1.0
Total cholesterol (mmol/L)	5.1 ± 0.8	5.3 ± 0.7
Triglyceride (mmol/L)	1.2 ± 0.5	1.7 ± 0.8
CRP (mg/L)	0.8 ± 0.0	2.8 ± 1.8
Urinary isoprostane 8-iso PGF2α (baseline) (ng/mL)	53.7 ± 17.1	73.4 ± 9.6
Urinary isoprostane 8-iso PGF2α (24 h) (ng/mL)	85.0 ± 21.6	91.7 ± 5.2

BMI = body mass index; CRP = C-reactive protein.

## Data Availability

All the data for this study will be made available upon reasonable request to the corresponding author.

## Supplementary Materials

The following are available online at https://www.mdpi.com/article/10.3390/cells10113109/s1, Figure S1: Proteins that did not differ between T2D and controls or within groups at differing timepoints, Figure S2: Negative correlation of HSPA1A with urinary isoprostane, Table S1: Inflammatory protein panel for all proteins for the type 2 diabetes patients, *p* value and fdr value (<0.05 fdr was considered significant).

## Abbreviations

HSPA1A	Heat shock 70 kDa protein 1A
HSPA8	Heat shock cognate 71 kDa protein
HSPD1	Heat shock protein family D (Hsp60) member 1
CDC37	Hsp90 co-chaperone Cdc37
CLU	Clusterin
DNAJB1	DnaJ homolog subfamily B member 1
MAPKAPK2	MAP kinase-activated protein kinase 2
MAPKAPK5	MAP kinase-activated protein kinase 5
PPID	Peptidyl-prolyl cis-trans isomerase D
STIP1	Stress-induced-phosphoprotein 1
TLR4:MD-2 complex	Toll-like receptor 4 in complex with MD-2
HSP90a/b	HSP90 dimer
CD274	Programmed cell death 1 ligand 1
UBE2L3	Ubiquitin-conjugating enzyme E2L 3
UBE2N	Ubiquitin-conjugating enzyme E2 N
UCHL1	Ubiquitin carboxyl-terminal hydrolase isozyme L1
AZU1	Azurocidin 1
CD40 ligand	Cluster of differentiation 40 ligand
IL34	Interleukin 34
IL5	Interleukin 5
RPS6KA5	Ribosomal protein S6 kinase 5
TBK1	TANK-binding kinase 1
PRKCZ	Protein kinase C zeta type
FGF8	Fibroblast growth factor 8
HMGB1	High mobility group box 1
IL10Rbeta	Interleukin 10 receptor beta
