# Supplementary material for: Heat Shock-Related Protein Responses and Inflammatory Protein Changes Are Associated with Mild Prolonged Hypoglycemia
Source: Cells. 2021 Nov 10;10(11):3109. doi: 10.3390/cells10113109 (PMC8618421; doi:10.3390/cells10113109)
Supplement: Supplementary file 1 [file cells-10-03109-s001.zip › cells-1419856-supplementary.pdf]

Figure S1. Proteins that did not differ between T2D and controls or within groups at differing timepoints

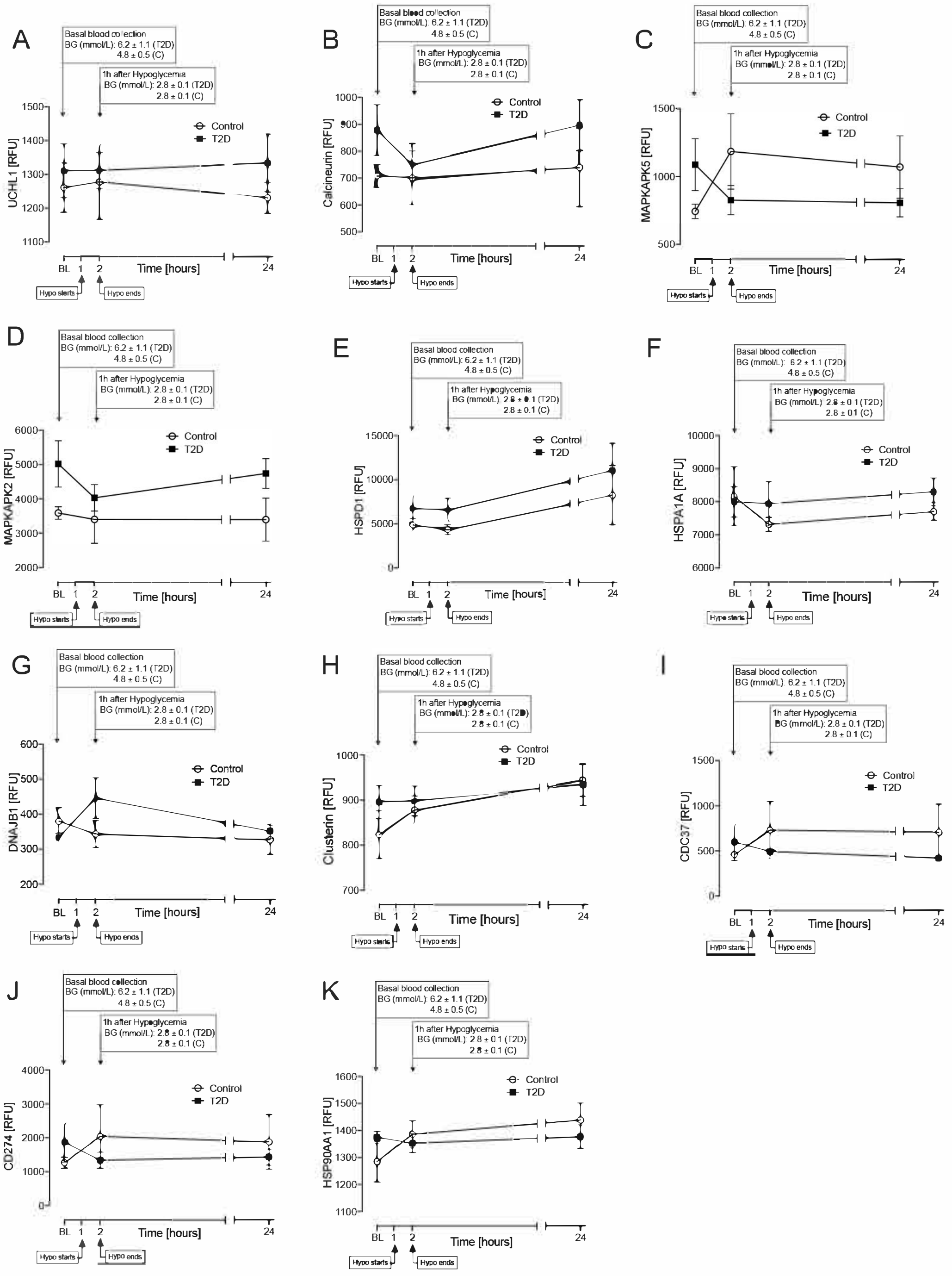

Figure S2. Negative correlation of HSPA1A with urinary isoprotane.

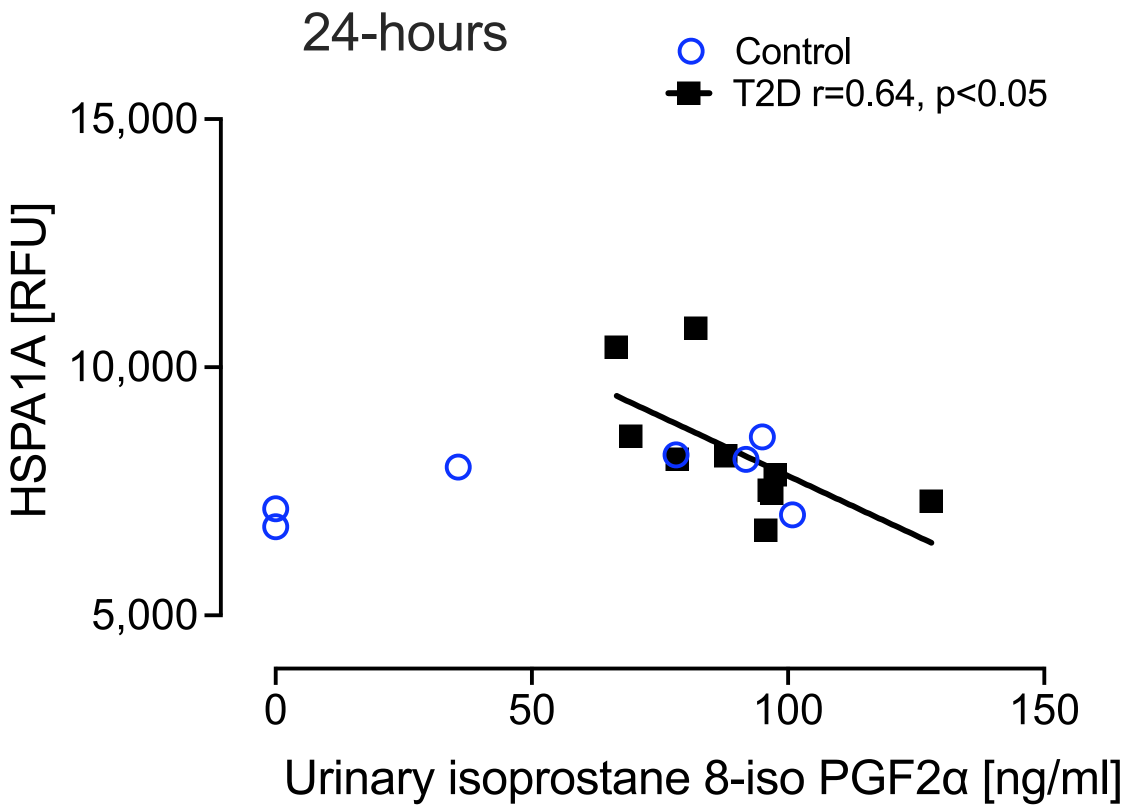

Table S1. Inflammatory protein panel for all proteins for the type 2 diabetes patients, p value and fdr values (<0.05 for fdr was considered significant) (17).

Target Full Name	P	fd
C-X-C motif chemokine 10	0.0003	0.0196
Interleukin-5	0.0003	0.0196
Azurocidin	0.0006	0.0268
C-type lectin domain family 7 member A	0.0008	0.0268
Serine/threonine-protein kinase TBK1	0.0012	0.0308
Protein kinase C zeta type	0.0017	0.0308
Ribosomal protein S6 kinase alpha-5	0.0017	0.0308
CD40 ligand	0.0017	0.0308
Interleukin-34	0.0020	0.0311
High mobility group protein B1	0.0022	0.0311
Protein S100-A9	0.0028	0.0361
Interleukin-1 beta	0.0041	0.0430
C-C motif chemokine 19	0.0042	0.0430
Sialoadhesin	0.0043	0.0430
Interleukin-10 receptor subunit beta	0.0047	0.0439
Fractalkine	0.0084	0.0745
Complement C3b, inactivated	0.0091	0.0754
C-X-C motif chemokine 5	0.0109	0.0851
Protein DJ-1	0.0115	0.0851
Tumor necrosis factor receptor superfamily member 11A	0.0147	0.1034
C-C motif chemokine 7	0.0175	0.1174
Interferon alpha-2	0.0249	0.1589
Tumor necrosis factor receptor superfamily member 19L	0.0259	0.1589
Lymphotactin	0.0297	0.1744
C-C motif chemokine 20	0.0333	0.1802
Tumor necrosis factor receptor superfamily member 21	0.0346	0.1802
Tumor necrosis factor receptor superfamily member 11B	0.0352	0.1802
Toll-like receptor 4:Lymphocyte antigen 96 complex	0.0366	0.1802
Interleukin-37	0.0371	0.1802
C-C motif chemokine 1	0.0383	0.1802
Interleukin-17B	0.0411	0.1869
C-C motif chemokine 15	0.0494	0.2178
C-C motif chemokine 4-like	0.0516	0.2206
Interleukin-23 receptor	0.0532	0.2206
Transforming growth factor beta-1	0.0623	0.2448
Growth-regulated alpha protein	0.0662	0.2448
Advanced glycosylation end product-specific receptor, soluble	0.0663	0.2448
Prostaglandin G/H synthase 2	0.0673	0.2448
Toll-like receptor 2	0.0677	0.2448

Oxidized low-density lipoprotein receptor 1	0.0718	0.2530
Insulin-like growth factor-binding protein 4	0.0797	0.2741
C-C motif chemokine 3-like 1	0.0908	0.3050
Phosphatidylinositol 4,5-bisphosphate 3-kinase catalytic subunit alpha isoform:Phosphatidylinositol 3-kinase regulatory subunit alpha complex	0.0946	0.3103
Tumor necrosis factor receptor superfamily member 1B	0.0969	0.3106
Interleukin-17D	0.1235	0.3793
Lymphocyte antigen 86	0.1264	0.3793
EGF-like module-containing mucin-like hormone receptor-like 2	0.1264	0.3793
Interleukin-6	0.1468	0.4313
Eotaxin	0.1678	0.4758
Endothelial monocyte-activating polypeptide 2	0.1687	0.4758
Interleukin-2 receptor subunit alpha	0.1725	0.4770
Interleukin-23	0.1831	0.4964
Tumor necrosis factor receptor superfamily member 1A	0.1970	0.5160
C-C motif chemokine 24	0.1976	0.5160
C-C motif chemokine 3	0.2030	0.5204
Tumor necrosis factor receptor superfamily member 9	0.2126	0.5354
Tumor necrosis factor receptor superfamily member 8	0.2183	0.5400
Interleukin-1 receptor-like 2	0.2284	0.5554
Hepatitis A virus cellular receptor 2	0.2374	0.5628
Interleukin-22	0.2431	0.5628
Lysozyme C	0.2479	0.5628
CD5 antigen-like	0.2504	0.5628
C-reactive protein	0.2515	0.5628
C-C motif chemokine 14	0.2578	0.5680
C3a anaphylatoxin des Arginine	0.2656	0.5687
Ras-related C3 botulinum toxin substrate 1	0.2684	0.5687
C5a anaphylatoxin	0.2702	0.5687
P-Selectin	0.2751	0.5704
Interleukin-10	0.2812	0.5747
C-C motif chemokine 13	0.2913	0.5789
Ck-beta-8-1	0.2927	0.5789
Complement C3	0.2956	0.5789
C-C motif chemokine 18	0.3089	0.5921
Thrombospondin-1	0.3167	0.5921
Interleukin-17F	0.3234	0.5921
Tumor necrosis factor receptor superfamily member 18	0.3245	0.5921
Interleukin-8	0.3252	0.5921
C3a anaphylatoxin	0.3276	0.5921
Complement C3b	0.3357	0.5921
C-C motif chemokine 2	0.3359	0.5921
Kininogen-1	0.3491	0.6077
Tyrosine-protein kinase HCK	0.3582	0.6160
Complement C3d fragment	0.3739	0.6313
C-X-C motif chemokine 6	0.3761	0.6313

Protein kinase C theta type	0.3962	0.6511
C-C motif chemokine 21	0.4014	0.6511
C-C motif chemokine 25	0.4054	0.6511
MAP kinase-activated protein kinase 2	0.4118	0.6511
Interleukin-27	0.4153	0.6511
C-C motif chemokine 17	0.4192	0.6511
C-X-C motif chemokine 13	0.4202	0.6511
C-C motif chemokine 22	0.4628	0.7030
Interleukin-13	0.4637	0.7030
PSA:alpha-1-antichymotrypsin complex	0.4750	0.7121
Tumor necrosis factor	0.4828	0.7121
C-X-C motif chemokine 11	0.4850	0.7121
Bone morphogenetic protein 6	0.4936	0.7121
Neutrophil-activating peptide 2	0.4961	0.7121
C-C motif chemokine 8	0.5000	0.7121
Tumor necrosis factor receptor superfamily member 4	0.5145	0.7228
Retinoic acid receptor responder protein 2	0.5177	0.7228
Peroxiredoxin-5, mitochondrial	0.5280	0.7270
Interleukin-17A	0.5351	0.7270
Tumor necrosis factor receptor superfamily member 10A	0.5375	0.7270
Macrophage colony-stimulating factor 1	0.5414	0.7270
Tumor necrosis factor receptor superfamily member 14	0.5558	0.7364
Alpha-1-antichymotrypsin	0.5642	0.7364
CD27 antigen	0.5660	0.7364
Macrophage colony-stimulating factor 1 receptor	0.5735	0.7364
Phosphatidylinositol 4,5-bisphosphate 3-kinase catalytic subunit gamma isoform	0.5791	0.7364
Calcium/calmodulin-dependent protein kinase type 1D	0.5830	0.7364
Connective tissue-activating peptide III	0.5850	0.7364
Natural cytotoxicity triggering receptor 3	0.5961	0.7396
Tumor necrosis factor receptor superfamily member 3	0.5980	0.7396
C-C motif chemokine 23	0.6254	0.7618
Complement C5b-C6 complex	0.6267	0.7618
Platelet factor 4	0.6351	0.7653
CD97 antigen	0.6429	0.7683
E-Selectin	0.6499	0.7701
Mast/stem cell growth factor receptor Kit	0.6754	0.7879
Complement C4b	0.6762	0.7879
Macrophage migration inhibitory factor	0.6930	0.8009
Annexin A1	0.7091	0.8129
Interleukin-1 alpha	0.7208	0.8196
Complement C4	0.7453	0.8406
C-C motif chemokine 16	0.7713	0.8631
Interleukin-1 Receptor accessory protein	0.7827	0.8690
Interleukin-18 receptor accessory protein	0.8035	0.8771
Complement C5	0.8075	0.8771

Tyrosine-protein kinase Lyn, isoform B	0.8087	0.8771
Allograft inflammatory factor 1	0.8225	0.8811
Sphingosine kinase 1	0.8249	0.8811
Extracellular matrix protein 1	0.8390	0.8895
Tumor necrosis factor receptor superfamily member 25	0.8612	0.9062
Tumor necrosis factor ligand superfamily member 4	0.8930	0.9327
Tumor necrosis factor receptor superfamily member 6B	0.9051	0.9370
Carbohydrate sulfotransferase 2	0.9104	0.9370
C-C motif chemokine 5	0.9285	0.9466
Tyrosine-protein kinase Lyn	0.9331	0.9466
Group IIE secretory phospholipase A2	0.9576	0.9644
Tumor necrosis factor-inducible gene 6 protein	0.9710	0.9710
